# The triennial International Pigment Cell Conference (IPCC)

**DOI:** 10.1186/s12967-018-1609-1

**Published:** 2018-10-03

**Authors:** Neil F. Box, Lionel Larue, Prashiela Manga, Lluis Montoliu, Richard A. Spritz, Fabian V. Filipp

**Affiliations:** 1 Department of Dermatology and Epidemiology, University of Colorado Denver, Aurora, CO USA; 2 CNRS, Equipe Labellisée Ligue Contre le Cancer, Normal and Pathological Development of Melanocytes, UMR 3347, Institut Curie, Orsay, France; 3 Ronald O Perlman Department of Dermatology, New York University Langone Medical Center, New York, NY USA; 4 CNB-CSIC, CIBERER-ISCIII, Centro Nacional de Biotecnología, Campus de Cantoblanco, Madrid, Spain; 5 Human Medical Genetics and Genomics Program, University of Colorado Denver, Aurora, CO USA; 6 Systems Biology and Cancer Metabolism, Program for Quantitative Systems Biology, University of California Merced, 5200 North Lake Road, Merced, CA 95343 USA

**Keywords:** Melanocyte, Melanin, Melanoma, Melasma, Albinism, Vitiligo, Oncology, Immunotherapy, Dermatology, Pigment, Pigmentation, Color, Skin, Translational medicine, Precision medicine, Cancer prevention, Conference, ICB, CPD, BRAF, MAPK, UV, IPCC, IFPCS

## Abstract

The International Federation of Pigment Cell Societies (IFPCS) held its XXIII triennial International Pigment Cell Conference (IPCC) in Denver, Colorado in August 2017. The goal of the summit was to provide a venue promoting a vibrant interchange among leading basic and clinical researchers working on leading-edge aspects of melanocyte biology and disease. The philosophy of the meeting, entitled Breakthroughs in Pigment Cell and Melanoma Research, was to deliver a comprehensive program in an inclusive environment fostering scientific exchange and building new academic bridges. This document provides an outlook on the history, accomplishments, and sustainability of the pigment cell and melanoma research community. Shared progress in the understanding of cellular homeostasis of pigment cells but also clinical successes and hurdles in the treatment of melanoma and dermatological disorders continue to drive future research activities. A sustainable direction of the societies creates an international forum identifying key areas of imminent needs in laboratory research and clinical care and ensures the future of this vibrant, diverse and unique research community at the same time. Important advances showcase wealth and breadth of the field in melanocyte and melanoma research and include emerging frontiers in melanoma immunotherapy, medical and surgical oncology, dermatology, vitiligo, albinism, genomics and systems biology, precision bench-to-bedside approaches, epidemiology, pigment biophysics and chemistry, and evolution. This report recapitulates highlights of the federate meeting agenda designed to advance clinical and basic research frontiers from melanoma and dermatological sciences followed by a historical perspective of the associated societies and conferences.

## Introduction—program highlights of the XXIII IPCC

The XXIII IPCC encompassed a series of opening and award lectures, including the IFPCS Presidential lecture and honorary lectures by the recipients of the Fitzpatrick, Lerner, and Seiji Awards. These awards are bestowed by regional pigment cell societies to leading international scientists who have made extraordinary contributions to pigment cell research. There were 8 plenary sessions and 32 concurrent sessions accomplishing a balance between clinical and basic melanoma and dermatology research.

In addition to opening lectures, the first day included a plenary session on the evolution of pigmentary systems across the animal kingdom and three concurrent sessions on advances in vitiligo, melanocyte, and melanoma biology as well as disease management. On the second day, two plenary sessions covered fascinating advances in the genetics of coloration in various animal species and in UV light responses and DNA repair. The fourth day included two plenary sessions and four concurrent sessions that emphasized basic biology of melanoma and the latest developments in translational melanoma therapies and resistance. Day five was headlined by a plenary session with keynote speakers on the impact of regenerative medicine on pigmentary diseases and a plenary session on the immunologic control of melanocytes and melanoma. General sessions covered evolutionary biology of pigmentation, developmental biology, genetics, genomic profiling, neuroscience and pigmentation, melanocyte stem cells and regenerative medicine, hair biology, mouse and non-mouse models of pigmentation, melanosome biogenesis and transfer, melanin function and chemistry, and UV signaling in the melanocyte. The program also included three lunchtime poster sessions with presentation of about 200 posters. Importantly, sessions were specifically dedicated to provide mentoring and career opportunities for young scientists, women in science, and underrepresented minorities in science.

The basic and clinical melanoma research sessions presented a state-of-the-art picture of immunotherapy, precision medicine, and genomic profiling of skin cutaneous, uveal, mucosal, and acral melanoma [1]. Keynote speaker Dr. Jeffrey Weber highlighted progress in melanoma management, while keynote speaker Dr. Douglas Brash presented the latest developments in DNA damage and melanoma risk with his work on UV-induced DNA damage and chemiexcitation of melanin. Frontiers of basic melanoma biology were introduced by keynote speaker Dr. Richard Marais who presented advances in targeted melanoma research integrating mouse models and therapies. The basic and clinical dermatology research sessions covered pigmentary processes, diseases, and treatments. The program featured keynote speakers including Dr. Sarah Tishkoff who addressed the genetic basis for skin pigmentation in African populations [2, 3]. Dr. Hopi Hoekstra identified the molecular basis of parental care evolution and mammalian striping phenotypes uncovered using the mouse as a model [4]. Dr. Rudolf Jaenisch provided insights into the forefront of mammalian embryonic and induced pluripotent stem cell research demonstrating the fundamental rules of cellular reprogramming [5]. Dr. Dennis Roop discussed progress on differentiating human induced pluripotent stem cells into keratinocytes, fibroblasts, and applications for skin grafting including melanocytes [6].

Highlights included updates on precision dermatology, bench-to-bedside approaches, genome-wide association studies, genetic diversity of pigmentation, and progress in immunotherapy [7]. Dr. Ian Jackson presented the analysis of the UK Biobank cohort of over 0.5 million genotyped individuals including detailed phenotype self-reports on pigmentation and hair color. This analysis allowed construction of detailed polygenetic models for blonde and red hair color, and penetrance assessment of many of the low frequency melanocortin 1 receptor (*MC1R*) variants. Dr. Nicolas Hayward delivered analysis of whole-genome sequences from cutaneous, acral and mucosal subtypes of melanoma. In contrast to frequently occurring coding and non-coding mutations in cutaneous melanoma, the Australian consortium resolved structural changes and novel signatures of mutagenesis in acral and mucosal melanomas, not previously identified in melanoma [8].

A number of presentations also explored the genetic diversity of pigmentation in non-human and non-mouse systems. Dr. Cheng-Ming Chuong presented a novel foray into the world of feather pigment pattern formation, where color patterns important for animal behavior and speciation is modulated by the presence, arrangement, or differentiation of melanocytes [9]. Among many other important clinical advances that were introduced during the meeting, Dr. Robert Andtbacka presented final results of a phase II multicenter combination trial that included ipilimumab and oncolytic virus immunotherapy [10]. Teams of Dr. Frank Meyskens and Dr. Stephane Rocchi elucidated the pivotal role of metabolic and transcriptional reprogramming in the switch of melanoma cells toward an invasive and drug-resistant phenotype [11, 12]. Dr. Jeffrey Weber and Dr. Dirk Schadendorf pointed to the importance of cancer-germline antigens that predict resistance to therapy response [13].

### Historical perspective of IFPCS and PASPCR unfolding the purpose of the IPCC—mission, vision, and future needs

**Fig. 1:**
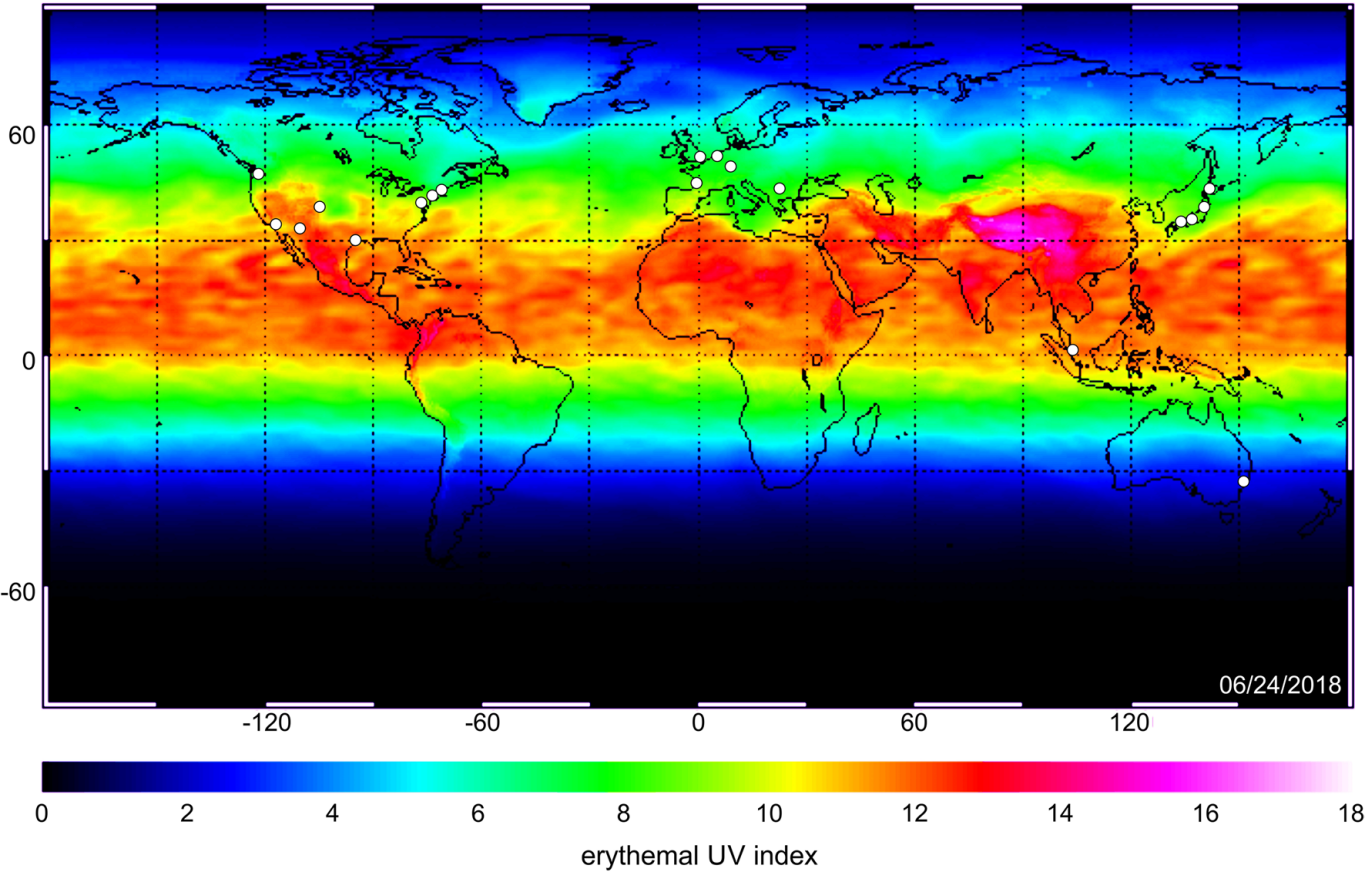
Historical locations of the triennial International Pigment Cell Conference (IPCC). Venues of the IPCC between 1946 and 2020 are indicated in white circles onto a world map of the erythemal UV index. The UV index is a measure for the effective UV irradiance and susceptibility to sunburn (1 unit equals 25 mW/m^2^) reaching the Earth’s surface recorded by satellites of the European Space Agency (clear sky on 24th July 2018)

The International Pigment Cell Conference has emerged as the sole international scientific convention devoted to the study of the normal pigment cell and the advancement of basic, translational, and clinical research on diseases involving pigment cells. The first meeting was held in New York in 1946 and reconvenes on a triennial basis (Fig. 1). Recent sites for this important international gathering have been Singapore (2014), Bordeaux (2011), and Sapporo (2008) (Table 1). While each meeting has had its own unique focus, commonalities for over 70 years have always been melanin, pigment cells and melanoma.

**Table 1:** Venues and award recipients of the International Federation of Pigment Cell Societies (IFPCS) at the triennial International Pigment Cell Conference (IPCC)

IPCC	Years	Location	Myron Gordon award	Seiji memorial lecture	Raper medal	Takeuchi medal	Aaron B. Lerner lecture	Fitzpatrick medal
					ESPCR	JSPCR	PASPCR	PCMR
I	1946	New York, NY, USA						
II	1949	New York, NY, USA						
III	1951	New York, NY, USA						
IV	1957	Houston, TX, USA						
V	1961	New York, NY, USA	G. A. Swan					
VI	1965	Sofia, Bulgaria	T. B. Fitzpatrick					
VII	1969	Seattle, WA, USA	A. B. Lerner					
VIII	1972	Sydney, Australia	E. J. MacDonald, V. J. McGovern					
IX	1975	Houston, TX, USA	V. Riley					
X	1977	Cambridge, MA, USA	H. S. Mason, G. Prota					
XI	1980	Sendai, Japan	M. Seiji					
XII	1983	Giessen, Germany	A. Anders, F. Anders, W. Quevedo	T. B. Fitzpatrick				
XIII	1986	Tucson, AZ, USA	J. T. Bagnara, M. E. Hadley	G. Prota				
XIV	1990	Kobe, Japan	Y. Mishima	A. B. Lerner				
XV	1993	London, UK	P. A. Riley, H. Rorsman, T. Takeuchi	T. Takeuchi	J. M. Pawelek			
XVI	1996	Anaheim, CA, USA	J. Matsumoto	R. A. King	G. Prota	V. J. Hearing	S. Orlow	
XVII	1999	Nagoya, Japan	V. J. Hearing, S. Ito	D. C. Bennett	K. Jimbow	G. Barsh		
XVIII	2002	Egmond aan Zee, The Netherlands	D. C. Bennett	V. J. Hearing	A. Thody	M. Tachibana, S. Shibahara		
XIX	2005	Reston, VA, USA	M. Mizoguchi	S. Shibahara	S. Ito	C. Goding	G. Barsh	
XX	2008	Sapporo, Japan	Kowichi Jimbow	Greg Barsh	J. C. Garcia-Borron	J. M. Pawelek	A. Slominski	Heinz Arnheiter
XXI	2011	Bordeaux, France	Zalfa Abdel Malek, Ruth Halaban, Shin-ichi Nishikawa	Richard A. Spritz	Marco d’Ischia	Yasushi Tomita	Coling Goding	Takahiro Kunisada
XXII	2014	Singapore, Malaysia	Greg Barsh	Richard Sturm	Jean-Paul Ortonne	Richard A. Spritz	William Pavan	Fabian V. Filipp
XXIII	2017	Denver, CO, USA	Colin Goding, Emi Nishimura	Shosuke Ito	Lionel Larue	Kazumasa Wakamatsu		Marie Webster, Ashani Weeraratna

**Fig. 2:**
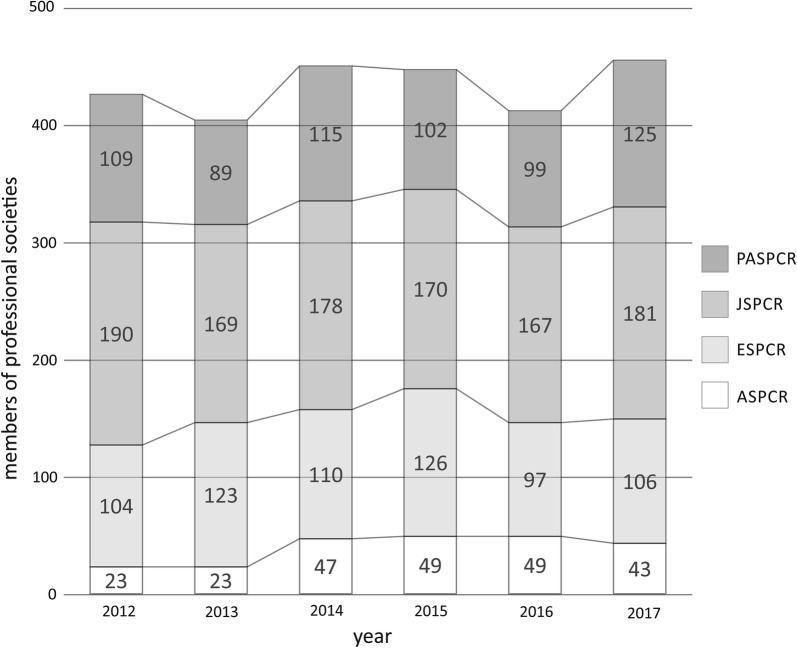
The composition of the International Federation of Pigment Cell Societies (IFPCS) and membership contribution from Pan-American Society for Pigment Cell Research (PASPCR), Japanese Society for Pigment Cell Research (JSPCR), European Society for Pigment Cell Research (ESPCR), and Asian Society for Pigment Cell Research (ASPCR)

In 1977, the International Federation of Pigment Cell Societies (IFPCS) was developed first as the International Pigment Cell Society and then formalized as IFPCS in 1990. With the IFPCS formally acting as a means to provide interaction between the independently formed sister societies, the IPCC meeting was chartered as a main function of IFPCS. The Japanese Society for Pigment Cell Research (JSPCR) was founded in 1984, the European Society for Pigment Cell Research (ESPCR) in 1985, the Pan-American Society for Pigment Cell Research (PASPCR) in 1988, and the Asian Society for Pigment Cell Research (ASPCR) in 2004, each becoming members of IFPCS at that time. Thus, the mission of the IFPCS is to disseminate cutting edge research via the publication of the federation’s journal, *Pigment Cell and Melanoma Research*, and to host the IPCC meeting. The IPCC remains the primary vehicle to promote worldwide scientific interchange for those international investigators who use an array of approaches to study pigment cell function in normal biology and disease. An international forum to present data, to discuss ideas, to identify key areas of needed research and to set new research directions is essential to ensuring the future of this vibrant, diverse and unique research community. The success of the IPCC to play this role in the pigment cell community is evidenced by the fact that attendance has been on the rise (Fig. 2).

### Recapitulation of the XXIII IPCC

The XXIII IPCC, representing the four member Pigment Cell Societies of the International Federation, was hosted by the Pan-American Society for Pigment Cell Research in Denver, Colorado, on 26–30 August 2017. 540 registrants from 28 countries participated in the 8 plenary and 32 concurrent sessions. 8 keynote speakers led the program, along with 21 plenary session speakers and 80 invited speakers. A further 103 speakers were chosen from submitted abstracts. The main program was complemented by four pre-conference satellite symposia and four pharmaceutical company sponsored symposia. The annual meeting of the Melanoma Prevention Working Group (MPWG) was hosted for the first time at the IPCC meeting. The MPWG functions under the umbrella of the Southwest Oncology Group (SWOG) and the Eastern Cooperative Oncology Group (ECOG). The MPWG meeting promoted clinically relevant research focused on key aspects of melanoma prevention and discussed innovative paths forward into clinical trials of chemoprevention agents for high-risk melanoma patients [14]. The comprehensive scientific program of the IPCC 2017 contained an equal weight of basic and clinical research in pigmentary diseases and melanoma. The importance of studying pigment cells is defined most dramatically by recent advances in melanoma therapies that can be lifesaving, but also by the many diseases and conditions intersecting with the pigmentary system that remain in need of effective treatments.

A major program highlight was a dedicated session on advances in basic and clinical research on albinism. Albinism is a rare genetically inherited condition affecting approximately one in twenty thousand people in most world populations. Albinism occurs at relatively high frequency in sub-Saharan Africa. People with albinism experience visual deficits, hypopigmentation, and sun-sensitivity. In less advanced societies, people with albinism may not have access to healthcare specialists, sunscreen, or protective clothes. Moreover, in sub-Saharan Africa, a humanitarian crisis is presently underway affecting patients with this disease [15]. The dedicated session on albinism raised awareness to this important issue, and sent a message of solidarity to patients suffering from albinism, leaving a unique footprint in the field of pigment cell biology.

### Planning and organization of IPCC 2017—special format, challenges, sustainability

IPCC 2017 was planned for late summer, 26–30 August 2017 in downtown Denver, Colorado. The strategy for designing and marketing of IPCC 2017 was informed by multiple lines of reasoning. First, with new melanoma therapies providing improvements in medical care, the field has progressed in an increasingly clinical direction, in some ways diminishing the focus on the basic research that has given rise to successful treatment strategies. Inevitably, new strategies will be needed to further advance the treatment of melanoma patients, and without a strong basic research base, these advances will not be possible. Moreover, successes in the translational arena of melanoma need to be duplicated in other key areas of pigment cell research, including vitiligo, melasma, and other pigmentary diseases. Providing a strong conference venue to support basic research in pigmentary diseases and melanoma in essence embraced the true mission of the IPCC meeting and of the IFPCS organization. A second influencing factor in designing IPCC 2017 was the recognition that with the World Melanoma Congress (WMC) associated with the Society for Melanoma Research (SMR) being scheduled for Brisbane in October, the IPCC would be the only major melanoma meeting to be held in the Northern Hemisphere in 2017. Thus a strong focus on melanoma would allow North American clinicians and researchers proximal access to the latest advances in the field. The third factor embraces all who are interested in pigment cell biology and disease by providing a comprehensive format for promoting interchange between such diverse groups of researchers. Thus, the theme of IPCC 2017 was strategically chosen as *Breakthroughs in Pigment Cell and Melanoma Research*. In essence, IPCC 2017 was at once an outreach to all members of the community and a call to stand unified in facing potential funding challenges as a field.

Initially, a dual focus on pigmentary disease and melanoma seemed to allow maximal opportunity for industry based meeting support. With strong support from the melanoma committee, major pharmaceutical company support was obtained to support the melanoma program. However, it proved more challenging to raise support from companies with an interest in pigment cell function and disease. This was compensated for by outstanding institutional support from groups within the University of Colorado system affiliated with the local organizing committee. Consequently, the meeting was supported by the melanoma related pharmaceutical industry, the University of Colorado, by conference registration fees and even through generous support from speakers, session chairs and attendees.

## Conclusions and outlook

### IFPCS and the IPCC as its global forum

The IFPCS is a vital global umbrella organization for the regional pigment cell societies. Over more than seven decades, the triennial IPCC has been an opportunity for scientist across the globe to gather to discuss recent findings, progress, and ongoing research. Consistently, the federation and local societies have provided the international research community with essential resources on all aspects of pigment cells including development, cell and molecular biology, genetics, diseases of pigment cells including melanoma.

### Joint Montagna-PASPR Symposium 2018 and IPCC 2020

In 2018, the Montagna Symposium on the Biology of the Skin and the Annual meeting of the Pan-American Society for Pigment Cell Research will host a joint conference entitled *Melanoma to Vitilio: The Melanocyte in Biology and Medicine* at Glendan Beach, OR commencing on October 17–22, 2018. The IPCC 2020 will be hosted in Yamagata, Japan from 18–21 July 2020. The title and focus of the XXIV triennial IPCC will be *Integration of Basic Science and Clinical Practice in Pigment Cell Biology*.

## Abbreviations

IFPCS: International Federation of Pigment Cell Societies
IPCC: International Pigment Cell Conference
JSPCR: Japanese Society for Pigment Cell Research
ESPCR: European Society for Pigment Cell Research
PASPCR: Pan-American Society for Pigment Cell Research
ASPCR: Asian Society for Pigment Cell Research
MPWG: Melanoma Prevention Working Group
SWOG: Southwest Oncology Group
ECOG: Eastern Cooperative Oncology Group
WMC: World Melanoma Congress
SMR: Society for Melanoma Research

## References

[1] Filipp FV, Birlea S, Bosenberg MW, Brash D, Cassidy PB, Chen S, D’Orazio JA, Fujita M, Goh B, Herlyn M (2018). Frontiers in pigment cell and melanoma research. Pigment Cell Melanoma Res.

[2] Crawford NG, Kelly DE, Hansen MEB, Beltrame MH, Fan S, Bowman SL, Jewett E, Ranciaro A, Thompson S, Lo Y (2017). Loci associated with skin pigmentation identified in African populations. Science..

[3] Martin AR, Lin M, Granka JM, Myrick JW, Liu X, Sockell A, Atkinson EG, Werely CJ, Moller M, Sandhu MS (2017). An unexpectedly complex architecture for skin pigmentation in Africans. Cell.

[4] Bendesky A, Kwon YM, Lassance JM, Lewarch CL, Yao S, Peterson BK, He MX, Dulac C, Hoekstra HE (2017). The genetic basis of parental care evolution in monogamous mice. Nature.

[5] Cohen MA, Markoulaki S, Jaenisch R (2018). Matched developmental timing of donor cells with the host is crucial for chimera formation. Stem Cell Rep.

[6] Kogut I, McCarthy SM, Pavlova M, Astling DP, Chen X, Jakimenko A, Jones KL, Getahun A, Cambier JC, Pasmooij AMG (2018). High-efficiency RNA-based reprogramming of human primary fibroblasts. Nat Commun.

[7] Filipp FV (2017). Precision medicine driven by cancer systems biology. Cancer Metastasis Rev.

[8] Hayward NK, Wilmott JS, Waddell N, Johansson PA, Field MA, Nones K, Patch AM, Kakavand H, Alexandrov LB, Burke H (2017). Whole-genome landscapes of major melanoma subtypes. Nature.

[9] Cooke TF, Fischer CR, Wu P, Jiang TX, Xie KT, Kuo J, Doctorov E, Zehnder A, Khosla C, Chuong CM, Bustamante CD (2017). Genetic mapping and biochemical basis of yellow feather pigmentation in budgerigars. Cell.

[10] Ribas A, Dummer R, Puzanov I, VanderWalde A, Andtbacka RHI, Michielin O, Olszanski AJ, Malvehy J, Cebon J, Fernandez E (2017). Oncolytic virotherapy promotes intratumoral T cell infiltration and improves anti-PD-1 immunotherapy. Cell.

[11] Ohanna M, Cerezo M, Nottet N, Bille K, Didier R, Beranger G, Mograbi B, Rocchi S, Yvan-Charvet L, Ballotti R, Bertolotto C (2018). Pivotal role of NAMPT in the switch of melanoma cells toward an invasive and drug-resistant phenotype. Genes Dev.

[12] Zecena H, Tveit D, Wang Z, Farhat A, Panchal P, Liu J, Singh SJ, Sanghera A, Bainiwal A, Teo SY (2018). Systems biology analysis of mitogen activated protein kinase inhibitor resistance in malignant melanoma. BMC Syst Biol.

[13] Shukla SA, Bachireddy P, Schilling B, Galonska C, Zhan Q, Bango C, Langer R, Lee PC, Gusenleitner D, Keskin DB (2018). Cancer-germline antigen expression discriminates clinical outcome to CTLA-4 blockade. Cell.

[14] Jeter J, Bowles T, Curiel-Lewandrowski C, Swetter S, Filipp FV, Chu E, Kirkwood J, Funchain P, Gershenwald J, Geskin L (2018). Chemoprevention agents for melanoma: a path forward into phase III clinical trials. Cancer.

[15] Franklin A, Lund P, Bradbury-Jones C, Taylor J (2018). Children with albinism in African regions: their rights to ‘being’ and ‘doing’. BMC Int Health Hum Rights.

